# Consequences and Mechanisms of Noise‐Induced Cochlear Synaptopathy and Hidden Hearing Loss, With Focuses on Signal Perception in Noise and Temporal Processing

**DOI:** 10.1002/advs.202409322

**Published:** 2025-04-07

**Authors:** Hui Wang, Steven J Aiken, Jian Wang

**Affiliations:** ^1^ Shanghai Key Laboratory of Sleep Disordered Breathing Department of Orolaryngology‐Head and Neck Surgery Otolaryngology Institute of Shanghai Jiao Tong University Shanghai Sixth People's Hospital Affiliated to Shanghai Jiao Tong University School of Medicine Shanghai P. R. China; ^2^ School of Communication Sciences and Disorders Dalhousie University Halifax NS Canada

**Keywords:** coding‐in‐noise deficits, noise‐induced synaptopathy, noise‐induced hidden hearing loss, ribbon synapses, temporal processing deficits

## Abstract

Noise‐induced synaptopathy and relevant hidden hearing loss (NIS and NIHHL) have been a hot topic in hearing research for almost 15 years. The progress is summarized in this review to address the reversibility of the synaptic damage after the initial loss, and the role of functional deficit in the repaired synapses as the reason for hearing impairment in addition to the deafferentiation caused by the synaptic loss, per se. The evidence supporting the synaptic repair is summarized. It is pointed out that coding‐in‐noise deficit (CIND) may not be the major problem of NIS and NIHHL, since solid evidence supporting the existence of this deficit is not available even in animal studies, as well as in in human reports. Rather, temporal processing deficits are clearly demonstrated in subjects with NIS and potentially NIHHL. The idea of CIND as the major concern in NIHHL is proposed based upon the functional categorization of the auditory nerve (ANF) by spontaneous rate and the biased loss of the ribbon synapses innervation the low‐SR ANF. The limitation of this hypothesis is discussed in detail. The review also addresses the difficulty of translating animal data to humans and the need for new research in the future.

## Graphical abstract

Noise‐induced synaptopathy (NIS) is largely reversible due to self‐repair. NIS and noise‐induced hidden hearing loss are two concepts with similarities and differences. The major hearing deficits in NIHHL are temporal processing disorders. The translation of animal data in NIS studies to humans is hindered by many factors.

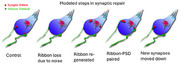

## 1 Introduction

Noise‐induced hearing loss (NIHL) is one of the most common neurological disorders, which is a sensorineural type of hearing loss resulting primarily from cochlear damage.^[^ ^1^ ^]^ The National Institute for Occupational Safety and Health has identified NIHL as one of the ten leading occupational diseases and injuries. Non‐occupational noise exposure also affects the hearing of children, youth, and adults.^[^ ^2^ ^]^ It is estimated that ≈1/3 of all cases of hearing loss (HL) in adults are related to noise exposure.^[^ ^3^ ^]^

NIHL is quantified as threshold shifts occurring after noise exposure, which can include both temporary threshold shifts (TTS) and permanent threshold shifts (PTS). In occupational safety control practice, only noise‐induced PTS (NIPTS) is used to quantify NIHL.^[^ ^4^ ^]^ However, the validity of this approach has been called into question by studies showing massive losses of afferent synapses between inner hair cells (IHCs) and spiral ganglion neurons (SGNs) in rodents even when a noise exposure causes no PTS, as first reported in CBA mice^[^ ^5^ ^]^ and later in Guinea pigs,^[^ ^6^ ^]^ rats and chinchillas,^[^ ^7^ ^]^ rhesus monkeys,^[^ ^8^ ^]^ and other strains of mice.^[^ ^9^ ^]^ In many of the reports cited above, noise‐induced synaptic loss was considered to be the major pathology of (NIS).

When NIS occurs without PTS, there is no NIHL according to the traditional definition and current standard. However, NIS may nevertheless impair suprathreshold hearing function; such a deficit of suprathreshold hearing function would be called a noise‐induced hidden hearing loss (NIHHL). It has been hypothesized that the primary deficit in NIHHL is difficulty hearing in background noise.^[^ ^10^ ^]^ This hypothesis is mainly based on two lines of research evidence. Firstly, single units of type I afferent auditory nerve fibers (ANFs) synapsing with inner hair cells (IHCs) are typically divided into 3 groups based on their spontaneous rates (SR) and other associated functional features. Low‐SR units have variable thresholds and larger dynamic ranges, allowing them to encode sound at high levels.^[^ ^11^ ^]^ Second, the coding of the low‐SR fibers to signal transients is more resistant to masking by background noise.^[^ ^12^ ^]^ Third, however, synapses between IHCs and low‐SR ANFs are more likely to be destroyed by noise and therefore may be non‐functional in cases of NIS without PTS.^[^ ^6^, ^13^ ^]^ Therefore, the selective loss of the low‐SR is considered as the major cause of signal coding at suprathreshold levels, especially in the masking by background noise. In a recent review of our group, we addressed the role of selective loss of low‐SR ANFs in the NIS and NIHHL and the limitations of the ANF categorization based upon SR in signal coding.^[^ ^14^ ^]^

The first report of massive synaptic loss without PTS after a single brief noise exposure^[^ ^5^ ^]^ was seen as particularly concerning from a health and occupational safety perspective because the noise‐induced synaptic loss was found to be irreversible in the strain of mice tested (CBA/Caj mice). Such an irreversible loss would likely result in a significant functional decline. If this were also the case in humans, then defining NIHL solely on the basis of threshold shifts would no longer be justifiable. However, these concerns have been somewhat alleviated by more recent evidence that synaptic losses are reversible in most species (see review in section 2 below), as well as by a lack of evidence that NIS causes coding deficits or hearing difficulties in noise. This challenges the hypothesized model of impairment based on damage limited to low‐SR ANFs.

In this review, evidence that establishes the reversibility of noise‐induced synaptic damage will be summarized. This will be followed by a discussion challenging the hypothesis that hearing in noise deficits are the primary functional impairments associated with NIS and NIHHL, followed by evidence showing that temporal processing deficits are more likely to be the primary consequence of NIS. We will then review the development of functional evaluations of NIHHL in association with NIS, as well as limitations and future directions. Although synaptopathy‐like changes have been reported in the central auditory system, the focus of the present review will be on the NIS in cochleae.

Similar to other types of hearing loss, NIS and NIHHL also lead de‐afferentiation to auditory brain and change of central gain. Many research papers have linked NIS/NIHHL with tinnitus and hyperacusis, which have been covered by excellent reviews published recently^[^ ^15^ ^]^ and therefore will not be the focus of the present review.

## 2 Noise‐Induced Cochlear Synaptic Loss Is Largely Reversible

Noise‐induced synaptic loss was explored in the 1990s, mainly by Pujol's group.^[^ ^16^ ^]^ In those early studies, however, the cochlear structure was mainly observed with electron microscopy and was not quantitative due to technical limitations. Since no damaged synapses were found in samples taken several weeks after noise exposure, it was concluded that synaptic damage was fully reversible.

The first quantitative study of synaptic loss after a brief (but not PTS‐inducing) noise exposure was done in CBA/Caj mice. In this strain, a dramatic loss of ribbon synapses (≈50%) in the high‐frequency half of the cochlea was found to be permanent.^[^ ^5^ ^]^ The permanency was supported by a similar amount of SGN degeneration quantified 64 weeks later, suggesting a loss of neurotrophic support of SGNs from IHCs and supporting cells as the result of the synaptic loss. Based on these results from CBA/Caj mice, it became a dominant assumption in the field that noise‐induced synaptic loss was permanent, although evidence emerged indicating that noise‐induced synaptic damage was reversible in reports from Wang's group in Guinea pigs^[^ ^6^ ^]^ as well as C57 and Kunming mice.^[^ ^9a,b^ ^]^ In the ensuing years, the massive noise‐induced synaptic loss was also reported in other species, such as rats, chinchillas,^[^ ^7^ ^]^ and rhesus monkeys.^[^ ^8^ ^]^ However, these studies only observed synaptic loss at a one‐time point following the noise exposure and thus could not speak to the issue of reversibility. During that time period, only one paper from researchers outside of Wang's group supported the reversibility of noise‐induced synaptic loss, based on results from C57 mice.^[^ ^9e^ ^]^

The story largely changed when two reports were published in 2019 with clear evidence of synaptic recovery in C57 mice after a massive initial loss.^[^ ^9c,d^ ^]^ Since then, the reversibility of noise‐induced synaptic damage has been gradually accepted by hearing science researchers. More recently, a report from Liberman's group provided a detailed comparison of synaptic changes in CBA/Caj mice and C57BL/6J mice after an initial noise‐induced loss.^[^ ^17^ ^]^ It is interesting to note that CBA mice have a deficit in synapse repair, unlike C57 mice and Guinea pigs. Generalization based on early reports from this particular strain of mice led to incorrect assumptions about the permanence of noise‐induced synaptic loss, and it now appears likely that noise‐induced synaptic loss is generally reversible, at least in part. Otherwise, most synapses would be quickly destroyed due to repeated noise exposure in life.

The reversibility of noise‐induced loss of ribbon synapses is supported by several lines of evidence in addition to synaptic counts via immunohistology staining. This research has been comprehensively reviewed^[^ ^18^ ^]^ and will be summarized below.

### 2.1 Morphological evidence of synaptic repair and plasticity after initial loss

The first evidence of synaptic repair was from transmission electron microscopy (TEM) images from the lab where noise‐induced synaptic damage was first reported^[^ ^16^, ^19^ ^]^ Synapses with extremely swollen post‐synaptic terminals but near‐intact presynaptic ribbon structures were seen shortly after noise exposure, while normal synaptic structure was seen weeks later. The damaged synapses would not have been counted as lost on the basis of immunohistology staining. Since synaptic count did not decrease further after the initial loss observed shortly after the noise exposure,^[^ ^5^, ^6^, ^9^ ^]^ it is reasonable to postulate that the damaged synapses were repaired.

Although the TEM measures listed above did not provide synapse counts to demonstrate recovery, several features have been noticed in connection to synapse regeneration. These include 1) afferent dendritic branches from single SGNs making multiple contacts with IHCs, 2) numerous efferent terminals that directly contact the IHC body instead of afferent terminals as normally seen in the cochleae of adult subjects,^[^ ^20^ ^]^ and 3) multiple presynaptic ribbons anchored to an elongated postsynaptic density (PSD).^[^ ^6^, ^20^ ^]^ Most of these features have been seen in immature cochleae during early development^[^ ^21^ ^]^ but not in mature cochleae except at the apical end.^[^ ^22^ ^]^ However, dendritic branches to multiple IHCs and multi‐ribbon synapses were reported in one study in the middle turn of adult mice cochlea.^[^ ^23^ ^]^ Further investigation is needed to verify if there is a quantitative increase in those features as a result of synaptic repair. Before such quantitative evaluation is completed, no clear conclusion can be drawn.

**Figure 1:**
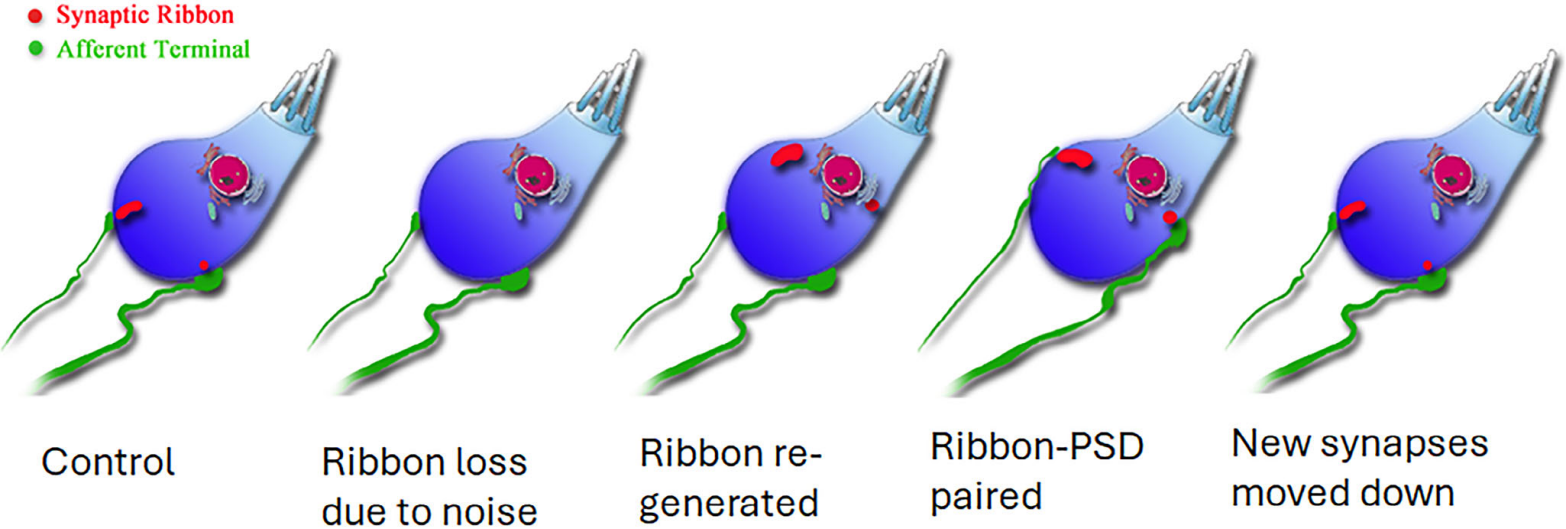
Dynamic location changes of afferent IHC synapses. Presynaptic ribbons are likely produced from ER and transported to the membrane near IHC nuclei. After being re‐established, synapses relocate to the bottom of IHCs. Reproduced with permission.^[^ ^6b^ ^]^. Copyright 2013, Shi et al.

Synaptic regeneration was also evidenced by position changes in some synapses after noise exposure. Normally, ribbon synapses are mostly located at the bottom of IHCs, although higher synaptic locations are seen at the modiolar side of IHC in mouse cochlea. In the studies in guinea pigs, significant changes in the position of the presynaptic ribbons were observed in IHCs shortly after noise exposure. Compared to the control cochlea, the mean location of the synaptic ribbons was higher and some of them were orphans (not paired with postsynaptic partners).^[^ ^6b^ ^]^ The distribution of ribbons returned to normal weeks later along with the recovery of the synaptic count. Based on this finding, Song et al. proposed an explanation, suggesting that shortly after noise exposure, new ribbons are assembled in the region of the endoplasmic reticulum (ER) around the nuclei, integrated with the nearby cell membrane to form new synapses with ANF terminals, and then repositioned to the bottom of IHCs (**Figure** **1**).^[^ ^6a^ ^]^ This hypothesized synaptic plasticity was supported by apparent neural fiber growth toward the region of the nucleus after noise damage, and observed in C57 mice but not in CBA mice,^[^ ^17^ ^]^ corresponding to the difference in synaptic regeneration abilities between the two strains. Furthermore, the dynamic positional change and recovery of synaptic ribbons that we reported have also been described in detail in association with ribbon protein metabolism in a very recent publication.^[^ ^24^ ^]^

### 2.2 Functional Data Supporting the Regeneration of Ribbon Synapses

**Figure 2:**
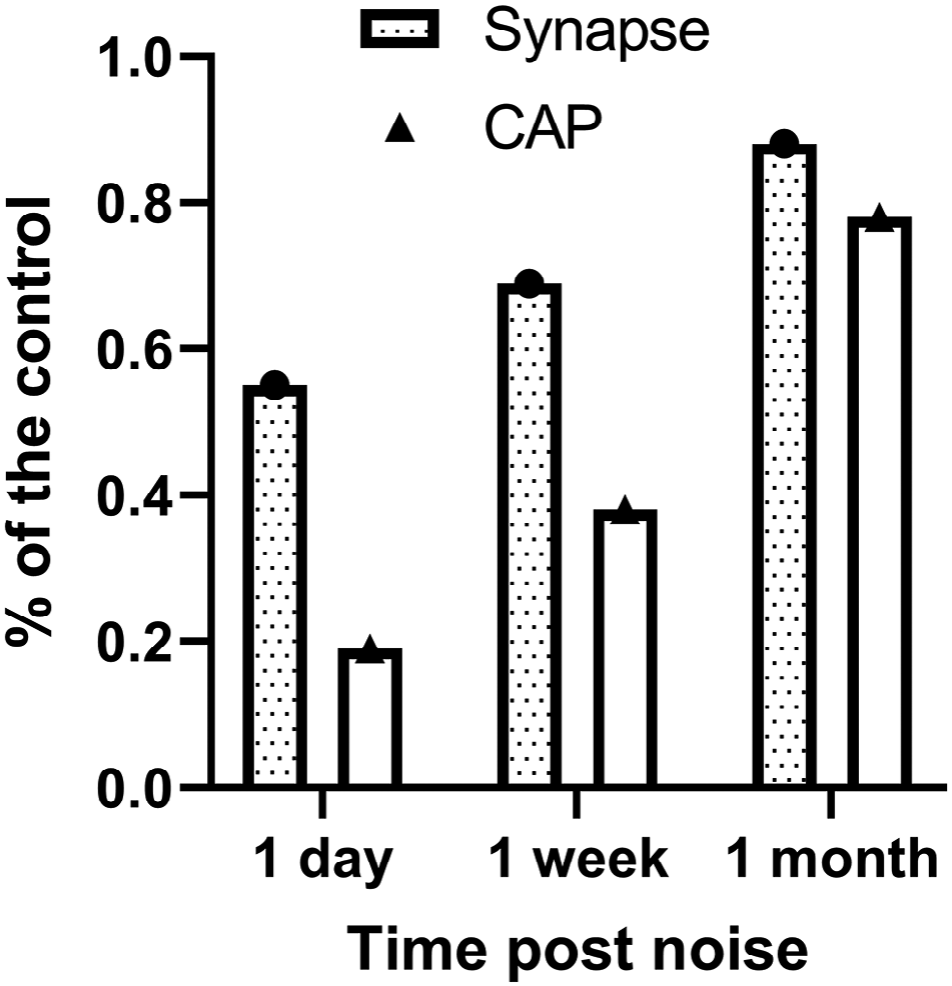
Normalized ribbon synapse count and maximal CAP amplitude after a noise exposure causing NIS without PTS in Guinea pigs.

In Guinea pigs, synapse regeneration is supported by a parallel recovery in the measured maximal amplitude of the compound action potential (CAP), which depends mainly on the number of functional ANFs (**Figure** **2**)^[^ ^6a^ ^]^ A partial recovery of wave I amplitude in the auditory brainstem response (ABR) was also reported in CBA mice^[^ ^5^ ^]^ but was attributed to threshold recovery by the authors, not synaptic regeneration since the recovery in ABR amplitude was completed in the same period of ABR threshold recovery. However, a role for synaptic regeneration in CAP amplitude recovery was supported by the fact that the increase in CAP amplitude largely occurred *after* the ABR threshold was fully recovered, a week after the noise exposure in guinea pigs.^[^ ^6b^ ^]^ The reduction in the CAP is much greater than expectation based upon the reduction in synaptic counts observed shortly after noise exposure (Figure 2). This is likely due to the combined effect of threshold elevation and the response change of single ANFs at this time point.^[^ ^6a^ ^]^ Even at one‐month post noise, CAP amplitude recovers less than the synapse count, suggesting that the surviving, repaired ANFs are still functionally weak.

The parallel recoveries of synapse count and threshold (although not fully synchronized) raise the possibility that synaptic repair is the mechanism of TTS recovery.^[^ ^9^, ^16^, ^19^, ^25^ ^]^ However, this conflicts with the hypothesis that noise‐induced reductions in auditory sensitivity are mainly due to damage to outer hair cells (OHC), which provide active gain for soft sounds.^[^ ^26^ ^]^ The dominance of OHCs in the threshold shifts over a 20–60 dB range has been demonstrated by the studies in which ABR and OAE are observed in the same animals (e.g., ref. [27]). Threshold recovery following TTS is associated with a full recovery of OHC function, demonstrated by a recovery of otoacoustic emissions (OAE)^[^ ^5^, ^28^ ^]^ and cochlear microphonics (CM).^[^ ^29^ ^]^ In addition, the repair of stereocilia and the tectorial membrane has been considered as a potential mechanism underlying the resolution of TTS in a number of studies.^[^ ^29^, ^30^ ^]^ Therefore, noise‐induced IHC‐SGN synapse damage and repair are less likely to be involved in threshold recovery, even if the synaptic damage involves ANF loss of all SR groups.^[^ ^31^ ^]^ Each IHC is innervated by more than 10 SGNs, and noise damage tends to be selective to synapses innervating high‐threshold fibers that have low spontaneous spike rates (SR),^[^ ^6^, ^13^ ^]^ similar to results obtained via ouabain‐induced cochlear damage at low doses.^[^ ^31^ ^]^ The idea that moderate damage to IHCs and their synapses with SGNs may not impact thresholds is also supported by the finding that up to a 60% loss of functional ANFs, due to selective IHC death induced by carboplatin in chinchillas, does not affect cochlear thresholds.^[^ ^32^ ^]^

Extant data cannot fully rule out changes in synaptic sensitivity that may occur in parallel with damage and repair of OHCs and surrounding structures. This possibility is supported by the hearing loss experienced by subjects with deficits in Otoferlin.^[^ ^33^ ^]^ The hearing loss caused by mutants of this gene appears mainly to be caused by dysfunction of ribbon synapses without functional abnormality of OHCs.^[^ ^34^ ^]^ Such cases of hearing loss serve as examples of threshold shifts with normal OHCs. In cases with OTOF mutations, malfunctions occur in all synapses and therefore all ANFs; while in the cases of NIS, the ANF synapses maintain normal sensitivity when there is no PTS due to OHC damage.^[^ ^6a^ ^]^

Functional evidence supporting synaptic regeneration was also found in a single‐unit study of ANFs in Guinea pigs, which showed that signal coding deficits had a much later onset (see below), than the initial synaptic loss.^[^ ^6a^ ^]^ It was hypothesized that prior to synaptic repair, only ANFs that remained connected to IHCs via surviving synapses contributed to the recording, and those ANFs were functionally near normal; while the coding behaviors averaged from ANFs recorded weeks later had contributions from ANFs connected to IHCs via regenerated synapses. Therefore, the later decline in coding function reflected the functional deficits of the repaired synapses.^[^ ^35^ ^]^ In other words, coding deficits may occur as the result of unhealthy synapse repair. In a very recent study, post‐noise synaptic regeneration in animals was further confirmed and was associated with declined binaural hearing.^[^ ^36^ ^]^

### 2.3 Synaptic Regeneration is Supported by Intrinsic Repair Mechanisms

The third line of evidence comes from the existence of an intrinsic mechanism for synaptic repair/regeneration mediated by neurotrophic factors. Neurotrophic factors naturally exist in IHCs and supporting cells (see review of ref. [37]). Their intrinsic role in the cochlea must be to maintain or repair neuronal structures, which likely includes the promotion of synapse repair after damage. This was proved by pharmacological experiments summarized below. CBA mice receiving exogeneous neurotrophin‐3 (NT‐3) via round window membrane shortly after noise exposure showed higher synaptic counts than controls,^[^ ^38^ ^]^ as has also been found in Guinea pigs.^[^ ^39^ ^]^ The repair‐promoting effect of NT‐3 in CBA mice is interesting because this strain of mice appears to lack the ability to self‐repair synapses disrupted by noise.^[^ ^5^, ^17^ ^]^ Similar results were reported in a study using a gene knock‐in mouse model to produce NT‐3 overexpression in supporting cells,^[^ ^40^ ^]^ and by gene transfection to IHCs with an adeno‐associated viral (AAV) vector.^[^ ^37^ ^]^ It is clear that synaptic repair is a therapeutic target with potential for clinical translation.

## 3 Hearing‐In‐Noise Deficits May Not Be The Primary Consequence Of Nihhl

### 3.1 Conceptual clarification of the differences between NIS and NIHHL

It is worth clarifying that NIS refers to synaptic damage as well as functional deficits related to the damage and subsequent repair, not only to synaptic loss and functional changes due to the loss. In most of the literature, NIS has been discussed only in the context of synaptic loss and the effects of deafferentiation on ANFs, specifically to speculate selective deafferentiation of low‐SR ANFs. It is now clear that functional deficits seen in animals and human subjects with NIS are not only due to the loss of synapses and the associated deafferentiation of ANFs but also due to coding deficits in surviving and (especially) regenerated synapses.^[^ ^6a^ ^]^

**Figure 3:**
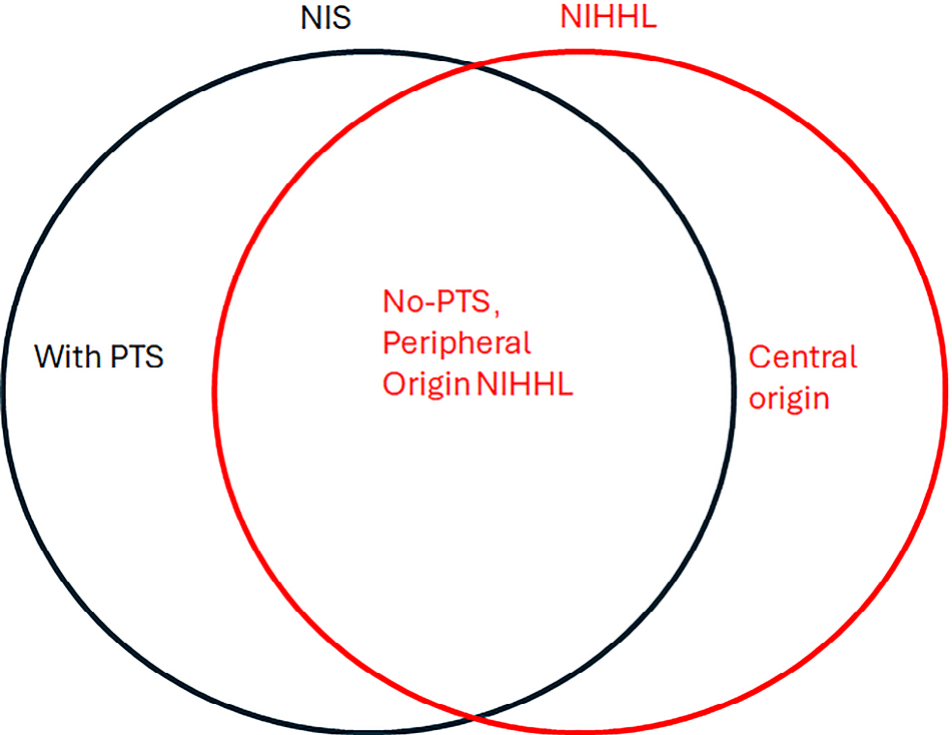
Conceptual overlaps and differences between NIHL and NIHHL. NIS is seen in NIHL with and without PTS. NIHHL is resulted from cochlear NIS without PTS and central contribution.

A relevant concept to NIS is NIHHL, which has been proposed to address the functional deficits associated with NIS without PTS. This concept was first coined by Schaette and McAlpine^[^ ^41^ ^]^ and has been widely accepted in the hearing research field.^[^ ^6^, ^10^, ^42^ ^]^ Hearing loss under such circumstances is hidden because it cannot be detected by routine audiological evaluations based on hearing thresholds. However, NIS can occur with and without a PTS. Therefore, the two concepts are overlapping but not 100% equal (**Figure** **3**). In addition, the discrepancy between the two terms is also due to the fact that, while NIS refers to a purely cochlear pathology, NIHHL may also have central origins (see review of ref. [37]).

### 3.2 Coding (or perception)‐in‐Noise Deficits are not Seen in Animal NIS Models

Currently, the primary hypothesized deficit in NIHHL is a difficulty with signal processing in high‐levels of background noise, or a coding(perception)‐in‐noise deficit (CiND or PiND). This idea is based on the finding that noise damage to IHC‐SGN synapses is biased to those innervating low‐SR ANFs. Two single‐unit studies in Guinea pigs demonstrated such a bias.^[^ ^6^, ^13^ ^]^ However, in the study with observations across multiple time points, the change of SR distribution related to the bias was found to be reversible with the repair of the synapses.^[^ ^6a^ ^]^ In the single unit study in CBA/Caj mice in which the synaptic loss was massive and permanent, ANF loss was found to be not limited to low‐SR units, as might be expected given the small size of this group relative to the total number of ANFs.^[^ ^43^ ^]^

The hypothesized CiND was based on the special role of low‐SR ANFs in signal coding. ANFs with low SR are considered more important for coding sound at high levels^[^ ^11a^ ^]^ and coding in the presence of noise masking.^[^ ^12^ ^]^ This is due to their high thresholds and larger dynamic ranges in their rate‐level functions (which are therefore less likely to be saturated), and their resistance to noise masking. In contrast, the rate‐level function of a typical high‐SR ANF is saturated at moderate sound levels, and therefore cannot encode amplitude changes of sounds at high levels.^[^ ^11a^ ^]^ Therefore, it is perfectly logical to postulate that CiND will be the main deficit associated with NIHHL.^[^ ^44^ ^]^

At this point in time, however, no solid evidence has been reported for CiND, even in animal models. There have been only a few single‐unit studies of NIS.^[^ ^6^, ^13^, ^43^ ^]^ However, none of these studies investigated CiND. There has been only one report that provided marginal evidence for hearing‐in‐noise deficits in rats using a pre‐pulse‐inhibition to startle response paradigm (PPI‐SR).^[^ ^42a^ ^]^ A narrowband burst of noise was presented in the background noise as the pre‐inhibitor. A reduced PPI to SR was seen in the noise‐exposed rats. However, this paradigm requires an inhibitor with a signal‐to‐noise ratio (SNR) of at least 20 dB, which is significantly higher than the SNRs used in behavior tests of speech‐in‐noise perception in humans. In such tests, subjects with normal hearing can typically understand speech at a 0 dB SNR.^[^ ^45^ ^]^ Moreover, the pre‐pulse inhibition of the startle responses involves the central auditory system, which may compensate for changes in cochlear function related to synaptopathy. Therefore, the results of this study are difficult to interpret with respect to the hypothesized impact of NIS on signal coding or perception in background noise.

Assessing cochlear responses to amplitude modulation (AM) with masking is a more direct and thus better way to evaluate CiND in animals and human subjects with NIS. Signals with AM are often used to evaluate signal processing in the auditory system. When recorded in the far field (e.g., as is done in clinical settings), they are typically called envelope‐following responses (EFRs). AM tones presented at higher intensities and lower modulation depths (e.g., 30%) theoretically challenge ANFs with high SRs, low‐thresholds and narrow dynamic ranges, since modulations may occur across levels at which the spike rate‐sound level functions of the ANFs are saturated, at least when masking is used to eliminate off‐frequency responses^[^ ^11^, ^46^ ^]^ With damage to low‐SR ANFs, one would thus expect to see a deficit in envelope following responses to such stimuli. However, in several EFR studies, no differences were found between subjects with NIS and controls after introducing masking,^[^ ^47^ ^]^ although the opposite result has also been found.^[^ ^46^ ^]^ Comparison of EFR results across different studies is difficult due to differences in methodology such as modulation frequency. On the other hand, a very interesting study showed that cortical internal noise was increased after ribbon synapses were damaged in mice via ouabain. The increased noise in the cortex could be the reason for subsequent hearing difficulties; such internal noise would cause hearing deficits no matter whether or not an external masker exists.^[^ ^48^ ^]^

One concern in animal studies of signal detection in noise is the masker type. In many laboratory studies using animal models, the maskers are stationary in level.^[^ ^18^, ^43^, ^47^, ^49^ ^]^ This does not reflect real‐world listening conditions where most maskers are temporarily fluctuating in amplitude. In such conditions, listeners often benefit from temporal modulations in the signal that provide access to speech information that would otherwise be masked (either energetically or via modulation masking^[^ ^50^ ^]^). It had long been found that subjects with poorer gap detection (a temporal processing ability) have significantly poorer speech‐in‐noise perception. Therefore, maskers used to evaluate CiND/PiND should also be temporarily fluctuating (see review of ref. [14]). However, even with the use of such a fluctuating masker, a recent study in Guinea pigs failed to demonstrate a significant difference between the noise‐exposed group and controls with respect to the effect of masking on the EFR.^[^ ^51^ ^]^

### 3.3 No clear association between hearing‐in‐noise deficits and NIS (in NIHHL) in humans

At this point, a clear connection between NIS and CiND/PiND in NIHHL has not been established in humans. A significant challenge in human studies of NIS is to obtain correct and reliable estimates of noise exposure and to select good control samples. One report examined speech perception in noise (within a battery of other tests) in 122 middle‐aged subjects (30‐57 years old) and found no clear link between noise exposure and speech perception.^[^ ^52^ ^]^ Negative results were similarly found in a study examining 148 young adults.^[^ ^53^ ^]^ In both studies, a reliance on self‐report to quantify noise exposure made it difficult to interpret the data.^[^ ^47b^ ^]^ In the review by Plack et al.,^[^ ^44^ ^b]^ the author stated that “Although the literature is not extensive, there is some evidence that listeners with a history of noise exposure, but with near‐normal threshold sensitivity, show deficits in complex discrimination tasks.” A small PiND was reported in several studies in subjects exposed to occupational noise. One early small‐sample study using workers with occupational noise exposure reported a 10% difference in the perception of high‐frequency word lists in noise between the noise‐exposed workers and the controls.^[^ ^54^ ^]^ However, the two groups were not audiometrically matched: the noise‐exposed group had a mean threshold that was 10 dB higher than that of the control group at 4kHz. In addition, the noise‐exposed group was 5 years older on average. More recently, a significant deficit in signal detection in noise was reported in subjects with occupational noise exposure in another small‐sample study with better matching of subjects between groups.^[^ ^55^ ^]^ Several other studies have also provided supporting evidence for CiND and/or PiND associated with NIS (NIHHL) in human subjects.^[^ ^56^ ^]^ However, evidence in those studies was not strong and some of them were focused on temporal processing rather than perception in noise,^[^ ^56b,c^ ^]^ and some studies also showed contamination from hearing loss (with threshold shifts) or brain injury.^[^ ^56a,b^ ^]^ On the other hand, many other studies have failed to find CiND/PiND in subjects with NIHHL.^[^ ^52^, ^57^ ^]^

The use of appropriate maskers is also a big concern in human studies, in which both stationary and temporarily fluctuating maskers (such as multi‐talker babble) have been used in speech‐in‐noise tests, although temporal feature of the maskers have not received careful consideration in research designs and there are no comprehensive comparisons of the masking effect from maskers with varying temporal features (see our previous review^[^ ^14^ ^]^). Rather, stationary noise has been used in some studies (e.g., ref. [53]) and other studies have used both stationary and fluctuating maskers but without comprehensive comparisons.^[^ ^56^, ^58^ ^]^

In human studies of PiND, another big challenge is potential interference from aging. The co‐existence of aging effects makes it difficult to identify the effects of noise exposure and attribute detected PiND to NIS. For example, the effect of age and noise exposure were evaluated in combination in an attempt to evaluate relative contributions.^[^ ^57a^ ^]^ The inclusion of age as a factor in this study made the identification of possible NIS effects more difficult. Since NIS under the umbrella of NIHHL may occur in combination with aging, the interpretation must consider the fact that cochlear synaptopathy is better verified in aging cochleae, where morphological evidence of SGN loss has been quantified post‐mortem in human temporal bones as a function of age.^[^ ^59^ ^]^ However, the effect of noise exposure was seen as a combined factor for increased synaptopathy with aging in human temporal bones.^[^ ^60^ ^]^

### 3.4 Challenge to the Functional Classification of ANFs by SR

The initial idea that NIS and NIHHL can be solely attributed to a selective loss of low‐SR ANFs has been challenged by the fact that noise‐induced damage and loss extends well beyond the small portion of ANFs in the low‐SR group^[^ ^5^, ^6^, ^8^, ^43^ ^]^ and by the fact that the effects of noise on the SR distribution of ANFs are reversible^[^ ^6a^ ^]^ with the regeneration of ribbon synapses, as has been confirmed by many studies.^[^ ^6^, ^9^, ^24^ ^]^ In addition to the facts above, the present lack of robust evidence for noise‐induced CiND/PiND calls into question whether the selective loss of low‐SR ANFs is the main reason for NIHHL as suggested by many reviews.^[^ ^10^, ^44^, ^57^, ^61^ ^]^ In a broader sense, this questions whether SR‐based classification is a critical mechanism of ANF coding of high‐level sounds (e.g., speech) in background noise, as suggested by classical reports.^[^ ^11^, ^12^, ^62^ ^]^

Although SR‐based classification is still widely accepted in the field as the basis for ANF coding of a wide range of sound levels, there are clear weaknesses in the theory, as summarized by an excellent review.^[^ ^63^ ^]^ It is now generally agreed that numerous mechanisms exist for the coding of auditory signals in cochleae by ANFs—especially for the coding of complex sounds at suprathreshold levels. Moreover, coding in the CAS likely involves more complex mechanisms that are beyond the scope of this review.

## 4 Nis Studies in Human Subjects: Challenges and Current Status

### 4.1 Concerns in Translating Animal Data to Humans

The concepts of NIS and NIHHL were proposed on the basis of dramatic synapse loss induced by a single, brief noise exposure at relatively high sound levels (100 dB SPL for mice and 106 dB SPL for Guinea pigs)—the maximal levels that do not cause PTS in those species. Such strong continuous (stationary) noise exposures are seldom experienced by humans. In industrial settings where such noises may exist, the exact noise level received is often controlled to be below 85 dBA, due to the presence of safety standards. In other non‐occupational situations (e.g., sports events, concerts, night clubs), noise levels are usually fluctuating or intermittent. In such cases, the patterns of exposure (periods of each exposure, total time, etc.) and the average level of sound need to be taken into consideration in order to understand the potentially damaging effects (see review of ref. [14]).

When lower sound levels are used, much less synaptic loss tends to occur, and the famous “equal energy” rule (used to predict noise‐induced cochlear damage) has not been found to be applicable to NIS. For example, one study observed synaptic loss in CBA mice after continuous exposure to 84 dB SPL noise for 168 h.^[^ ^64^ ^]^ Compared to the more commonly studied dosage of 100 dB SPL for 2 h, this noise exposure delivered much more total energy, as calculated using a 3 dB‐per‐doubling rate. However, the amount of synaptic loss was found to be far lower (<20% in the high‐frequency region, as compared to the 50% loss reported previously^[^ ^5^ ^]^). In a recent study using fluctuating noise below 90 dB SPL, to better mimic noise that might be experienced by humans, much less synaptic loss was also found in a Guinea pig model,^[^ ^51^ ^]^ even though the total exposure was equal to 106 dB SPL for 2 h (which caused a ≈50% loss of ribbon synapses^[^ ^6a^ ^]^).

### 4.2 The Lack of Morphological Evidence

It is almost impossible to identify noise‐induced synaptic damage or loss in human cochleae due to ethical restrictions. Limited post‐mortem cochlear analyses have found synaptic damage in subjects with normal hearing thresholds and OHCs.^[^ ^65^ ^]^ However, the synaptic losses in such samples cannot be fully attributed to noise exposure because of the confound of aging.^[^ ^59^ ^]^

### 4.3 The Lack of Reliable Functional Measurements for NIS Estimation

Another difficulty in investigating NIS in human subjects stems from the lack of measures that can be reliably associated with the quantity of synaptic loss and therefore the amount of deafferented ANFs. At present, several objective methods have been proposed for the detection of NIS in NIHHL in human subjects. A brief summary is provided below.

#### 4.3.1 Methods Using Evoked Transient Responses

Several objective methods based on transient evoked far‐field responses have been proposed for the detection of NIHHL in humans. In general, these methods aim to quantify reduced cochlear neural output to the auditory brainstem related to the loss/damage of synapses. ANFs will no longer respond to sound after a loss of synapses with IHCs, and ANFs connected via damaged synapses will reduce their firing response to sound,^[^ ^6a^ ^]^ as supported by the literature listed below.

- Reduction of wave I amplitude of the auditory brainstem response (ABR) has been observed in tinnitus subjects with^[^ ^57e^ ^]^ and without noise‐exposure history.^[^ ^66^ ^]^ Unfortunately, the small amplitude and large variation of wave I make this a difficult measure to use in clinical settings.
- Increase of the ABR wave V/I ratio. This is a measure of central gain. Since wave V is likely not to be reduced or even increased after peripheral damage, the ratio should be greater in subjects with HHL.^[^ ^44b^ ^]^
- Shifting of ABR peak latency by masking.^[^ ^49^, ^67^ ^]^
- Increased ratio of the summating potential (SP) to the compound action potential in ECochG.^[^ ^68^ ^]^

The use of these transient responses to estimate the loss of ribbon synapses and the deafferentation of ANFs is limited first by their response amplitude. In laboratory animals, CAPs recorded from round window electrodes can reach several hundred microvolts. Using such measurements, changes in CAP amplitude were found to be proportional to changes in synapse count.^[^ ^6a^ ^]^ However, far‐field measures such as the ABR suffer from small amplitudes and poor signal‐to‐noise ratios (SNR). Human ABR amplitude is typically below 0.5 µV, while the maximal amplitude of ABR from rodents is one order higher in magnitude. Therefore, it is generally difficult to use ABR amplitude value quantitatively in audiological clinics. However, in laboratory settings by experienced researchers, a significant correlation between wave I amplitude and performance in speech perception in noise in normal‐hearing subjects has been reported.^[^ ^69^ ^]^

ECochG generally shows larger response amplitudes than ABR, depending on the electrode montage employed. Currently, clinical ECochG recordings rarely employ optimal methods for obtaining responses with high SNRs (e.g., the use of needle or eardrum electrodes) since these tend to be uncomfortable and often require local anesthesia, as well as skill to place the needle or eardrum electrodes appropriately. The amplitude of the CAP or ABR wave I from the more commonly used ear canal ECochG electrode is only slightly larger than that recorded with a regular ABR electrode montage (e.g., with a reference electrode on the mastoid or earlobe).

Another concern with using transient evoked responses in the evaluation of noise‐induced synaptic loss and deafferentation comes from the need to evaluate the selective loss of synapses innervating low‐SR ANFs. It is generally recognized that transient responses are insensitive to this type of loss because low‐SR ANFs contribute to transient responses much less than high‐SR ANFs. This is due to their tendency not to fire reliably to stimulus onsets.^[^ ^31^ ^]^

Despite the limitations of transient responses discussed above, efforts to improve the usefulness of this approach for clinical use in the evaluation of NIS are ongoing. A recent mouse study reported that a curvature‐based quantification of wave I correlated more strongly with synaptopathy than a standard measurement of wave I amplitude and latency, and this was reported to be “robust and reliable”.^[^ ^70^ ^]^ The curvature method quantifies the degree of deviation of a curve (such as ABR wave I) from a straight line in order to quantify synaptopathy. The underlying reason for this is related to the fact that ABR wave I depends on the conduction speed and fiber diameter of contributing neurons.^[^ ^71^ ^]^ In a recent human study, curvature at the peak was found to be negatively correlated with age and behavioral thresholds in extended high frequency (EHF) regions.^[^ ^71^ ^]^ Although this method has shown some potential, more work needs to be done in humans with clearer indications of synaptopathy to validate its usefulness. A series of studies from Kelly Harris's group proposed some CAP metrics tested at high stimulus levels (70–110 dB pSPL) with the potential to identify age‐related synaptopathy.^[^ ^72^ ^]^ However, the usefulness of those CAP metrics in NIH evaluations remains to be validated. The studies from this group emphasized the link of LSR ANFs with aging.^[^ ^73^ ^]^ As discussed in this review, the selective loss of LSR ANF may not be the major contributor to NIH and NIHHL. This is consistent with the weak connection between noise exposure and the CAP changes tested at high sound levels in the attempt to explore the impact of LSR ANF loss.^[^ ^74^ ^]^

#### 4.3.2 Measuring HHL With Envelope Following Responses

Auditory neurons can synchronize their activity to the periodicity of external signals. The use of amplitude‐modulated (AM) signals is common practice in hearing research and in audiological clinical assessment. AM responses are typically called envelope‐following responses (EFR) or auditory steady‐state responses (ASSR)^[^ ^46^, ^75^ ^]^ because they follow periodic changes in the envelope as opposed to transient stimulus features. They generally have better frequency selectivity than transient responses based on clicks, chirps, or tone‐pips and reflect the contributions of neurons beyond onset responses.

EFRs (and the steady‐state responses (SSRs) in the broader sense) have been used for many different purposes, ranging from hearing threshold estimations^[^ ^76^ ^]^ to investigations of temporal processing.^[^ ^77^ ^]^ They have also been used for the diagnosis and quantification ANF deafferentation as a result of synaptic loss,^[^ ^46^, ^78^ ^]^ which is a major issue in cochlear synaptopathy. Modulation frequencies (MF) above ≈80 Hz give rise to EFRs dominated by contributions from peripheral and brainstem sources,^[^ ^46^, ^78^, ^79^ ^]^ although small contributions from the cortex may be involved up to ≈200 Hz,^[^ ^80^ ^]^ and myogenic contributions from the post‐auricular muscles may also be involved.^[^ ^81^ ^]^

Shaheen et al demonstrated the usefulness of EFR in detecting the deafferentation of ANFs in a CBA/caj mouse model, in which the ribbon synapse loss was found to be 50% or more at the 32 kHz CF region. Using AM with a carrier at this frequency, EFR amplitude was found to be reduced by 55%, nicely matching the amount of ANF deafferentation.^[^ ^46^ ^]^ In this study, the EFR was recorded in the far field with an electrode montage regularly used for non‐invasive ABR, and the modulation frequency (MF) varied from 400–1990 Hz with 100% modulation depth (MD). Using this set of parameters, the EFR was found to have a peak ≈1 kHz MF in control animals. Peak amplitude was greatly reduced in animals with synaptic loss (Figure 1). The noise‐induced reduction of the 1 kHz peak in the EFR‐MF curve (associated with the synaptic loss) suggests an ANF origin. The synchrony is surprisingly high considering the refraction time of the single ANFs. Likely the synchrony is achieved by the integration of phase‐locking across multiple ANFs (volley theory^[^ ^82^ ^]^).

In any case, the reduction of EFR amplitude by NIS suggests that this may be a useful method for evaluating NIHHL from synaptic loss or deafferentation. However, as pointed out by the authors, threshold elevation also reduces EFR. This means that the method can only be used in animals and human subjects with normal hearing thresholds. This makes it a less viable clinical tool for diagnosing synaptopathy when threshold shifts are also present. In addition, EFR amplitudes recorded in the mice were <0.3 µV. It would be expected to be much smaller in humans, particularly at higher modulation frequencies. This, and the associated poor SNR, limits the application of this method in humans. However, the advantages of EFR for detecting cochlear synaptopathy, which could be caused by various types of SNHL (e.g., ototoxicity, noise, and aging), have been recognized in comparison with responses to transient stimuli. Efforts have been made to optimize the methodology of measuring EFR,^[^ ^83^ ^]^ and some progress has been made, as is summarized below.

First, EFRs evoked by signals with different MFs can distinguish the contributions from CAS and auditory peripherals. It has been recognized that EFRs with low MFs contain a large contribution from CAS, the lower the MF, the more the central contribution. However, EFRs with MFs above 100 Hz can detect noise‐induced cochlear synaptopathy in humans.^[^ ^11^, ^69^, ^77^, ^78^, ^83^, ^84^ ^]^ EFR magnitude is quickly reduced with increasing MF and is difficult to use in humans.^[^ ^83b^ ^]^ However, some researchers found that EFR can be detected with 200 Hz MF with limited contribution from CAS^[^ ^79^, ^80^ ^]^ up to 500 Hz in young listeners.^[^ ^85^ ^]^

Secondly, EFR to rectangularly amplitude‐modulated (RAM) pure tones are more sensitive than EFRs obtained with conventional sinusoidally amplitude‐modulated (SAM) stimuli for the diagnosis of synaptopathy in humans.^[^ ^83^, ^86^ ^]^

Thirdly, RAM using 500‐ms, 4000 Hz tone bursts at 70 dB SPL has been found to have the potential for identifying synaptopathy associated with aging.^[^ ^83a^ ^]^ In this study, methodological details were investigated to optimize the outcome. Modeling analysis showed that the optimized measure was strongly reduced by synaptopathy (up to 85% amplitude reduction for an 87% loss in ANF) and reduced much less by reductions in cochlear gain due to OHC damage and loss (≈15%). The test‐retest reliability of the RAM‐EFR showed a ≈10% deviation of the median across sessions, which appears to be better than the SAM‐EFR (≈20% deviation of the median) as evaluated in young adults.^[^ ^83^, ^87^ ^]^ However, the overall amplitude of the RAM‐EFR is <0.2 µV, although it is larger than SAM‐EFR. Even with the RAM‐EFR, the SD is ≈0.04 µV or ¼ of the overall amplitude. These quantities should be taken into consideration when the method is used for diagnosis. The EFR measurement was initially proposed to provide sensitivity to potential noise‐induced selective loss of low‐SR ANFs, based upon the fact that, at high stimulus levels, high‐SR fibers are saturated and synchronize poorly to the envelope of AM signals.^[^ ^88^ ^]^ Therefore, EFR responses at the high stimulation level mainly reflect contributions from low‐SR fibers. If these fibers are not functioning properly (as the result of a noise‐induced selective loss of ribbon synapses connecting low‐SR ANFs), EFR responses at high levels will be reduced— as long as small modulation depths are used to ensure saturation of high‐SR ANFs.^[^ ^75b^ ^]^ Several factors must be taken into consideration when using this approach to investigate NIS. Firstly, low‐SR ANFs take a smaller portion of the total number of ANFs (≈16% in cats^[^ ^11a^ ^]^ and ≈10% in gerbils^[^ ^89^ ^]^) as compared to those with high SR. Secondly, noise‐induced synaptic loss is not limited to synapses innervating low‐SR ANFs in terms of the quantity of the synaptic loss.^[^ ^5^, ^6^ ^]^ Third, the SR distribution of ANFs likely returns to normal after repair and regeneration, although an SR bias is seen in the initial loss.^[^ ^6a^ ^]^ In light of these factors, EFR using high‐level signals with small modulation depths may not be important for the diagnosis of cochlear synaptopathy, at least in cases of NIS, unless the goal is to investigate the contribution of the low‐SR ANF loss to HHL.

Finally, it is worthwhile to point out that the above measurements of EFR amplitude aimed only to estimate the impact of synaptic loss (and ANF deafferentation) and not to assess possible signal processing deficits in ANFs connecting to IHCs via surviving and repaired synapses, since amplitude was the focus of analysis. Conceptually, however, cochlear synaptopathy is not limited to synaptic loss but also includes coding deficits of surviving and repaired synapses, such as temporal processing deficits.^[^ ^6^, ^15^, ^35^, ^77^, ^90^ ^]^

#### 4.3.3 Detecting cochlear synaptopathy by measuring the middle ear acoustic reflex

The middle ear muscle reflex (MEMR) has been investigated as a potential measure of cochlear synaptopathy likely related to noise exposure.^[^ ^91^ ^]^ In animal studies, the MEMR threshold has been found to be more sensitive to NIS than ABR wave I.^[^ ^92^ ^]^ Since MEMR is measured at sound levels well above hearing thresholds, it is a suprathreshold measurement by nature. The mouse study by Valero et al. used CBA/Caj mice and noise exposures similar to what was used by Kujawa and Liberman.^[^ ^5^ ^]^ MEMR was measured in mice that were awake to avoid the impact of anesthesia. This method is necessary because the MEMR is greatly reduced in anesthetized animals.^[^ ^92a^ ^]^ The potential involvement of the MOC was also ruled out by the use of KO mice. The MEMR was measured as a change of the probe sound level before vs. during the presentation of the elicitor in the contralateral ear: the activation of the MEMR by the elicitor increased middle ear impedance and therefore increased the SPL of the probe stimuli in the external ear.

The MEMR has been investigated as a tool for detecting cochlear synaptopathy in humans, but unfortunately, the evidence to date has failed to support its use. In one study, the MEMR was compared between young normal controls and those with tinnitus and speech perception‐in‐noise (SPiN) difficulties but normal audiometric thresholds^[^ ^57d^ ^]^— both were associated with noise exposure as verified via a structured interview. No evidence was seen for a connection between MEMR thresholds and either tinnitus or SPiN difficulty. In another study, the MEMR was found to be reduced in young Veterans as compared to the non‐veterans. However, the difference did not reach statistical significance.^[^ ^91c^ ^]^ The authors suggested that MEMR was relatively insensitive as compared with ABR and EFR measurements. In an effort to connect MEMR with speech perception ability in quiet and in noise in subjects without noise exposure, a negative result was also reported for the results in background noise (by QuickSIN).^[^ ^91b^ ^]^ In a more recent study comparing subjects with and without occupational noise exposure, no significant difference in the MEMR was found, suggesting the insensitivity of this measure in identifying NIS in humans.^[^ ^91a^ ^]^ Finally, another recent human study found that the MEMR was not associated with life‐span noise exposure.^[^ ^57j^ ^]^

## 5 Is Temporal Processing Deficit the Major Problem in NIHHL?

To date, most studies on the functional deficits in NIHHL have focused on the effects of ANF deafferentation, especially the loss of low‐SR ANFs. Here the present review will evaluate evidence of temporal processing deficits in terms of its existence and significance.

### 5.1 Temporal Coding Deficits Are Seen in Animals with NIS Without PTS

Temporal resolution is much higher in the auditory system as compared to the visual system.^[^ ^93^ ^]^ The synapse between IHCs and SGNs is the first speed‐limiting site of temporal processing along the auditory ascending pathway. This synapse is characterized as having a ribbon‐like structure in the presynaptic region of the synapse, which is widely recognized as a structure that facilitates the quick release of neurotransmitters across the synapse, which is critical for the synapse's high temporal resolution.^[^ ^42^, ^94^ ^]^ Any damage to these synapses may result in temporal coding deficits. This has been evidenced in Guinea pigs after a noise exposure of 105 dB SPL for 2 h that led to a massive loss of ribbon synapses (≈50% in the frequency above 8 kHz) but not a PTS. However, the synaptic loss was reduced to ≈18% one month later in this species. A temporal coding deficit was evidenced by 1) an increase of peak latency in the post‐stimulus time histogram (PSTH), 2) a reduced peak spike rate in the PSTH, and 3) an elongation of responses in a paired‐click forward masking paradigm.^[^ ^6a^ ^]^ Similarly, poor temporal processing was seen in CAP recordings using a paired‐click paradigm.^[^ ^6^ ^b]^ In both the single unit and the CAP study, the deterioration in temporal processing was seen after the threshold and synaptic counts were largely recovered, suggesting that the repaired synapses were malfunctioning. ABR evoked by paired clicks has also been used by others in detecting temporal processing changes in animals following NIS in rats.^[^ ^95^ ^]^

The impact of NIS on temporal processing has also been investigated with EFR in animal models. To address differences in the noise exposure experienced in human life from that used in typical NIS laboratory research, this study employed a fluctuating, intermittent noise exposure at a moderate level (below 90 dB SPL) to create NIS in Guinea pigs.^[^ ^51^ ^]^ In this study, temporal modulation transfer functions were measured in skull‐recorded EFRs at a moderate sound level (75 dB SPL) with varied modulation frequency. In such evaluations, a more rapid decrease in EFR with increasing modulation frequency was found in animals with NIS. Interestingly, the more rapid decrease was only significant in EFRs obtained with low‐modulation‐depth stimuli, suggesting that the temporal processing changes were mediated by low/medium‐SR units. Since the synaptic loss had been largely recovered in this study (with only a ≈6% loss of synapses at the time when the EFR was tested), it was postulated that the temporal processing deficits occurred in the repaired synapses.

The contribution of ANFs of different groups to temporal processing is complex. While low‐SR ANFs have weaker onset responses than high‐SR ANFs,^[^ ^31^ ^]^ they do contribute to temporal processing, especially at high sound levels. A modeling study suggested that the signal envelope is predominantly encoded at lower sound levels by high‐SR ANFs with characteristic frequencies (CF) near the carrier frequency of AM signal, but the signal envelope at higher levels is equally encoded by both low‐SR ANFs with CFs at the carrier frequency and high‐SR ANFs with CFs away from the carrier frequency.^[^ ^96^ ^]^ The model suggests that a healthy population of high‐SR ANFs be sufficient to encode the envelope over a wide level range. Currently, NIS is considered as predominantly affecting low‐SR ANFs, although this idea has been challenged.^[^ ^6^, ^14^, ^43^, ^47^ ^]^ Based upon the modeling results discussed above, any deficit in envelope coding due to a selective loss of low‐SR fibers could only be detected by using notched noise to mask the contribution from the high‐SR ANFs with CFs distal from the carrier frequency. In such a test, the SNR would have to be carefully selected.^[^ ^96^ ^]^ However, there is insufficient data from animal recordings to validate the modeling outcomes.

### 5.2 Temporal Processing Deficits in humans are associated with NIS and HHL

Temporal processing deficits have been reported in human studies of NIS (see reviews of refs. [14, 15]) and in old adults with normal hearing thresholds.^[^ ^97^ ^]^ In a small sample study, Stone et al. reported that subjects with exposure to recreational noise were poorer than controls in detecting temporal fluctuations of signals in background noise, even though noise‐exposed subjects were 7 years younger than control subjects on average.^[^ ^56c^ ^]^ Both groups had normal and equal pure‐tone thresholds from 2–4 kHz. However, this disadvantage was not seen at high sensational levels (SL) but appeared only when the signal was at low levels (≈12 dB SL). Although the results were interpreted in terms of subclinical IHC dysfunction, they were inconsistent with a theory of selective loss of high‐threshold, low‐SR nerve fibers as the major cause of functional deficits in NIHHL. In another study with a larger sample size, AM detection (for MFs between 60 and 200 Hz) was found to be poorer in train drivers (n = 28) than in 90 age‐matched controls^[^ ^56b^ ^]^ with stimuli presented at 80 dB SL. Negative results were also reported. For example, no change in binaural temporal processing was found to be associated with more lifelong noise exposure (identified by questionnaire).^[^ ^98^ ^]^ Since the measurement of the temporal processing was based on MEMR in this study, the negative result might have been due to the insensitivity of the method.

As reviewed previously, temporal processing is a mechanism that plays an important role in signal detection in background noise. This is supported by a comprehensive evaluation of the role of temporal processing in SPiN that was reported very recently.^[^ ^99^ ^]^ However, temporal processing deficits have not been recognized broadly in the field as a major perceptual consequence of NIS in NIHHL, nor in association with CiND/PiND (see representative reviews published very recently^[^ ^10^, ^15^, ^98^ ^]^). Consistent with the idea that temporal processing deficit may serve as the major deficit in NIHHL is the argument that many speech‐in‐noise tasks (such as QuickSIN) are not sensitive to HHL since they do not require a large reliance on temporal processing abilities associated with synaptic functions. This argument has been addressed by our previous reviews and a nice review by others.^[^ ^100^ ^]^

## 6 Summary of Past Research and Future Directions

After a long debate, it has been recognized that noise‐induced synaptic loss is largely reversible, although recovery is not 100%. This partial reversibility is consistent with the existence of intrinsic mechanisms for synaptic repair mediated by neurotrophins.^[^ ^101^ ^]^ In most animal studies of NIS, noise exposure has been induced by sound levels presented as high as possible without causing PTS. In the few studies that have used lower‐level noise exposures (<90 dB SPL), the synaptic loss has been found to be far lower, even when a higher total dose of noise has been applied.^[^ ^51^, ^64^ ^]^ When occupational safety regulations are enforced, noise exposure is seldom over 90 dB SPL in the workplace, except high‐level peaks that may occur, such as impulse noises (e.g., from jackhammers). The effect of such noises mimicking human noise exposure should be investigated in more detail using animal models to quantify the synaptic loss. Available data has shown that exposure to fluctuating, intermittent noise at lower levels results in much less synaptic loss.^[^ ^51^ ^]^ Moreover, a ‘toughening’ effect of pre‐exposure to low‐level noise has been found to greatly reduce synaptic loss.^[^ ^102^ ^]^ In consideration of all of these findings, the degree and impact of NIS on hearing is likely far lower than estimated on the basis of the initial animal study showing a ≈50% loss from a single, brief exposure. Therefore, synaptic loss found in laboratory animals cannot be easily translated to human beings due to species differences and, more importantly, differences in typical noise exposures.^[^ ^14^ ^]^

Currently, the dominant opinion is that NIS in the form of HHL impacts hearing via deafferentation following the synaptic loss, particularly due to a selective loss of low‐SR ANFs, leading to a deficit of hearing in noise. In line with this, many of the studies reviewed in this paper have focused on finding diagnostic tools appropriate for human use that are sensitive to synaptic loss. Significant noise‐induced deafferentation has not been verified in humans due to ethical considerations, with evidence for deafferentation limited to post‐mortem studies of temporal bones as a function of age.^[^ ^59^ ^]^ The quantification of physiological and functional consequences associated with noise‐induced deafferentation is still an important goal, but to date, there are no reliable evaluation tools that can identify deficits in humans that are clearly associated with NIS. While this awkward situation reflects the difficulty of diagnosing cochlear deficits in non‐invasive far‐field recordings and the difficulty of collecting accurate clinical information, another possibility needs to be considered: synaptic loss (or deafferentation) may not be the primary cochlear pathology induced by noise. Temporal processing deficits have been reported in human studies of NIS (see reviews of refs. [14, 15]) and in old adults with normal hearing thresholds.^[^ ^97^ ^]^ Although a few studies have shown temporal processing deficits in both animal and human subjects in association with noise exposure, much less research has focused on this than on the consequences of synaptic loss. Temporal processing should receive much more attention in future NIHHL research.

Finally, it has been recognized that noise damage to cochlear ribbon synapses, as shown in laboratory animals, is not limited to those innervating low‐SR ANFs, and SR‐based functional categorization has been challenged as the main contributor to the intensity coding by ANFs. However, functional diversity across ANFs is still an important mechanism for cochlear signal processing. This is evidenced by various diversities across synapses to single IHC that are correlated to ANFs with different functional features, including SR. The details of the diversities and their connection to the functional contribution to signal coding are currently being explored and research in this area is quickly moving forward based on many new technologies (see review ref. [103]). Future studies are needed to explore the change in the synaptic diversity induced by NIH and NIHHL and the impact of such changes on signal processing.

## Conflict of Interest

The authors declare no conflict of interest.
